# Effective Antibiotics in Combination against Extreme Drug-Resistant *Pseudomonas aeruginosa* with Decreased Susceptibility to Polymyxin B

**DOI:** 10.1371/journal.pone.0028177

**Published:** 2011-12-05

**Authors:** Tze-Peng Lim, Winnie Lee, Thean-Yen Tan, Suranthran Sasikala, Jocelyn Teo, Li-Yang Hsu, Thuan-Tong Tan, Nur Syahidah, Andrea L. Kwa

**Affiliations:** 1 Singapore General Hospital, Department of Pharmacy, Singapore, Singapore; 2 Changi General Hospital, Department of Laboratory Medicine, Singapore, Singapore; 3 Singapore General Hospital, Department of Infectious Disease, Singapore, Singapore; 4 Division of Infectious Diseases, Department of Medicine, Yong Loo Lin School of Medicine, National University of Singapore, Singapore, Singapore; Los Angeles Biomedical Research Institute, United States of America

## Abstract

**Objective:**

Extreme drug-resistant *Pseudomonas aeruginosa* (XDR-PA) with decreased susceptibility to polymyxin B (PB) has emerged in Singapore, causing infections in immunocompromised hosts. Combination therapy may be the only viable therapeutic option until new antibiotics become available. The objective of this study is to assess the *in vitro* activity of various antibiotics against local XDR-PA isolates.

**Methods:**

PA isolates from all public hospitals in Singapore were systematically collected between 2006 and 2007. MICs were determined according to CLSI guidelines. All XDR-PA isolates identified were genotyped using a PCR-based method. Time-kill studies (TKS) were performed with approximately 10^5^ CFU/ml at baseline using clinically achievable unbound concentrations of amikacin (A), levofloxacin (L), meropenem (M), rifampicin (R) and PB alone and in combination. Bactericidal activity (primary endpoint) was defined as a ≥3 log_10_ CFU/ml decrease in the colony count from the initial inoculum at 24 hours.

**Results:**

22 clinical XDR-PA isolates with PB MIC 2–16 µg/ml were collected. From clonal typing, 5 clonal groups were identified and nine isolates exhibited clonal diversity. In TKS, meropenem plus PB, amikacin plus meropenem, amikacin plus rifampicin, amikacin plus PB exhibited bactericidal activity in 8/22, 3/22, 1/22 and 6/22 isolates at 24 hours respectively. Against the remaining ten isolates where none of the dual-drug combination achieved bactericidal activity against, only the triple-antibiotic combinations of ARP and AMP achieved bactericidal activity against 7/10 and 6/10 isolates respectively.

**Conclusion:**

Bactericidal activity with sustained killing effect of ≥99.9% is critical for eradicating XDR-PA infections, especially in immunocompromised hosts. These findings underscore the difficulty of developing combination therapeutic options against XDR-PA, demonstrating that at least 3 antibiotics are required in combination and that efficacy is strain dependant.

## Introduction

Antimicrobial resistance is increasing worldwide and is of particular concern in gram-negative bacilli (GNB) where there is a paucity of new and effective antimicrobial agents [1]. *Pseudomonas aeruginosa* infections are associated with increased mortality and morbidity, especially in immunocompromised and burns patients respectively [2], [3]. The organism is capable of developing resistance to practically all classes of antibiotics and has always been considered a difficult target for antimicrobial chemotherapy [4]. Antibiotic resistance mechanisms in *P. aeruginosa* include inducible *amp*C beta-lactamases, efflux pumps (MexAB-OprM), loss of porin channels (OprD) and aminoglycoside-modifying enzymes [5]. Unfortunately, these mechanisms are often present simultaneously, thereby conferring extremely-drug resistant (XDR) phenotypes that are defined as non-susceptibility to at least one agent in all but two or fewer antimicrobial categories (i.e. bacterial isolates remain susceptible to only one or two categories) [6].

Antibiotic-resistant *P. aeruginosa* is a major concern in Singaporean hospitals. Up to 12.8% of bacteremia isolates are resistant to the carbapenems, while resistance rates for other commonly-used antibiotics such as amikacin, cefepime, ciprofloxacin and piperacillin-tazobactam range from 9.6% to 22.3% [7], [8]. In Singapore, polymyxin B (PB) is often prescribed as a last resort for XDR-*P. aeruginosa* (XDR-PA) infections. Unfortunately, the local susceptibility rate is only 66.7%, with the rest demonstrating intermediate resistance to PB [8]. This poses a serious challenge for clinicians as current evidence suggest that the clinically achievable level of polymyxins in the plasma is as low as 2.38 mg/L using present dosing regimens [9]. Until more data is available on the use of higher treatment doses, polymyxin monotherapy may be inadequate for treating a significant proportion of severe infections caused by XDR-PA, and antibiotic combination therapy may be the only viable option at present [10], [11].

The objective of this study was to assess the *in vitro* activity of various antibiotics individually and in combination against local strains of XDR-PA with decreased susceptibility to PB.

## Materials and Methods

### Sample collection

Clinical isolates of *P. aeruginosa* isolates were collected from six major Singaporean hospitals over a two-year period, beginning from February 2006. Each hospital was assigned a target number of isolates to collect (chosen to be representative of the relative size of the institution). Collection of each target organism began at the same time period, and continued until the assigned number of isolates was achieved. Only the first isolate was collected from each patient. The bacteria were stored at −70°C in Protect® (Key Scientific Products, Inc, Stamford, TX) storage vials. Fresh isolates were sub-cultured twice on 5% blood agar plates (Thermo Scientific Microbiology, Malaysia) for 24 h at 35°C prior to each experiment.

### Minimum inhibitory concentration testing

Minimum inhibitory concentrations (MIC) to piperacillin/tazobactam, cephalosporins (ceftazidime, cefepime), carbapenems (imipenem, meropenem & doripenem), aminoglycosides, (gentamicin, amikacin), aztreonam, ciprofloxacin, and PB were obtained using custom-made dehydrated microbroth dilution panels (Trek Diagnostics, West Sussex, UK) performed according to the manufacturer's recommendations. In brief, turbidity-adjusted bacterial suspensions from fresh overnight cultures were added to cation-adjusted Mueller-Hinton broth (Ca-MHB, BBL, Sparks, MD) to achieve an inoculum of 10^6^ CFU/ml. Minimum inhibitory concentrations for rifampicin and levofloxacin were obtained by macrobroth dilution [12]. Categorical susceptibility was based on Clinical Laboratory Standards Institute breakpoints except for rifampicin, for which there are no standard breakpoints [13]. XDR-PA isolates (defined as isolates with resistance to all tested antibiotics except PB) were used for subsequent experiments.

### Clonal relationship analysis

The clonal relatedness of the XDR-study isolates was assessed using repetitive-element-based PCR (rep-PCR) [14]. Genomic DNA was extracted using the PureLink™ Genomic DNA Mini Kit (Invitrogen Corp., Carlsbad CA) according to the manufacturer's instructions. PCR products were analyzed by chip-based microfluidic electrophoresis (Experion, Biorad, USA). Digitalized banding images were exported and cluster analysis was performed using Bionumerics 5.4 (Applied Maths, Kortrijk, Belgium). The isolates were considered as indistinguishable (similarity of DNA fragment pattern ≥90%) or distinct (similarity of DNA fragment pattern <90%).

### Presence of metallo-beta-lactamase

Phenotypic screening for metallo-beta-lactamases was performed by imipenem-EDTA double disk synergy method, as described previously [15]. Subsequently, a multiplex PCR assay was performed to determine to detect and differentiate the five major families of acquired metallo-beta-lactamase (MBL) in a single reaction [16]. Briefly, five primer pairs that were specific for each family of acquired MBLs were used to amplify the respective MBL gene fragments.

### Antimicrobial agents

Five drugs representing the major antibiotic classes were used in the study. Meropenem was provided by Astra Zeneca Inc. Amikacin, PB and rifampicin were purchased from Sigma-Aldrich (St. Louis, MO), while levofloxacin was provided by Daiichi Sankyo Co., Inc. For PB, meropenem, amikacin and levofloxacin, a stock solution of each antimicrobial agent in sterile water was prepared, aliquoted, and stored at −70°C. Prior to each susceptibility test, an aliquot of the drug was thawed and diluted to the desired concentrations with Ca-MHB. Rifampicin was dissolved in dimethyl sulfoxide and serially diluted to the desired final drug concentration [12]. The final dimethyl sulfoxide concentration had no effect on *P. aeruginosa* growth.

### Time-kill studies

Time-kill studies (TKS) were conducted with amikacin, meropenem, levofloxacin, rifampicin and PB alone and in combination. These antibiotics were chosen to represent each major antibiotic class with unique mechanisms of action. Utilizing the 5 antibiotics, a total of 10 possible two-antibiotic combinations were tested with all the isolates. If none of the two-antibiotic combinations achieve the pharmacodynamic endpoint as described below, we will test the isolate against a total of 10 possible three-antibiotic combinations. The concentrations chosen represent clinically achievable free or unbound plasma concentrations. For the purpose of our study, amikacin (a concentration-dependent antibiotic) was tested at 80 mg/L representing a free peak concentration arising from a once daily 2 g bolus dose [17]. The meropenem concentration of 64 mg/L represents a free peak concentration arising from a 2 g, 3-hour infusion dose [18]. Rifampicin (another concentration dependent antibiotic) was tested at 2 mg/L, representing a free peak concentration arising from a 600 mg oral or intravenous dose to maximize the utility of the drug [19]. The PB concentration of 2 mg/L represents the steady-state free serum concentrations achievable with at least 1 million units of PB [20].

Overnight bacterial cultures were diluted with pre-warmed Ca-MHB and incubated further at 35°C until log-phase growth was reached. The bacterial suspension was diluted with Ca-MHB according to absorbance at 630 nm. The final concentration of the bacterial suspension in each flask was approximately 10^5^ CFU/mL (ranging from 1×10^5^ CFU/ml to 5×10^5^ CFU/ml). The experiment was conducted in a shaker water bath set at 35°C. Serial samples (0.5 mL each) were obtained in duplicates from each flask at 0 (baseline), 2, 4, 8 and 24 hours and centrifuged at 10,000× *g* for 15 minutes followed by decantment. The pellets were then reconstituted with sterile normal saline to their original volumes in order to minimize drug carryover effect. Bacterial load was determined by quantitative culture, to determine the effects of various drug exposures on the total bacterial population over time. Total bacterial populations were quantified by spiral plating 10× serial dilutions of the samples (50 µl) onto Mueller-Hinton II agar plates (Thermo Scientific Microbiology, Malaysia). The plates were incubated in a humidified incubator (35°C) for up to 24 hours and the bacterial density from each sample was enumerated visually. The theoretical reliable lower limit of detection was 400 CFU/ml.

### Pharmacodynamic endpoints

For time-kill testing, we used bactericidal activity that was defined as a ≥3 log_10_ CFU/ml decrease in the colony count from the initial inoculum at 24 hours, as the primary endpoint. This endpoint is synonymous with sustained killing effect of ≥99.9%.

## Results

### Susceptibility studies

Twenty-two XDR-PA isolates were identified from 608 isolates from the urinary tract, blood and respiratory tract. PB MICs ranged from 2 to 16 mg/L while rifampicin MICs were ≥64 mg/L. 42% and 32% of the isolates were deemed to be PB resistant and intermediate respectively according to CLSI standards. The MICs of the various antimicrobial agents were shown in Table 1.

**Table 1:** Susceptibilities, MBL gene(s) detected and clonal grouping of XDR PA isolates.

			MIC (mg/L)					
Isolates	Clone	Amikacin	* Levofloxacin	Meropenem	*Rifampicin	PolymyxinB	MBL detected	#Other resistance genes detected
PA 403	6	≥128	≥64	≥64	≥64	**8**	VIM-like	-
PA 154	7	64	≥64	16	≥64	4	IMP-like	-
PA 386	5	≥128	≥64	≥64	≥64	**16**	VIM-like	-
PA 5	8	≥128	≥64	32	≥64	2	-	OXA 10
PA 6	9	32	≥64	≥64	≥64	2	IMP-like	-
PA 15	10	≥128	≥64	≥64	≥64	4	IMP-like	-
PA 30	3	64	≥64	32	≥64	2	-	-
PA 35	4	≥128	≥64	≥64	≥64	2	IMP-like	-
PA 28	5	64	≥64	8	≥64	4	-	-
PA 47	2	64	≥64	16	≥64	4	-	-
PA 48	1	≥128	≥64	16	≥64	**16**	-	-
PA 14	2	32	≥64	≥64	≥64	**8**	VIM-like	-
PA 14004	11	≥128	≥64	32	≥64	**16**		VEB 1
PA 425	12	64	≥64	32	≥64	4		VEB 1, OXA 10
PA 426	4	≥128	≥64	≥64	≥64	4	VIM-like	-
PA 377	5	≥128	≥64	≥64	≥64	**8**	VIM-like	-
PA 3355	3	≥128	≥64	≥64	≥64	**8**	VIM-like	-
PA 31165	13	64	≥64	≥64	≥64	4	IMP-like	-
PA 2854	1	64	≥64	≥64	≥64	2	VIM-like	-
PA 37428	5	≥128	≥64	≥64	≥64	2	IMP-like	-
PA 19224	5	≥128	≥64	≥64	≥64	**8**	-	-
PA 50116	5	16	≥64	≥64	≥64	**8**	IMP-like	-

*MICs were obtained using the macrobroth dilution method according to CLSI.

Bold type denotes resistant phenotypes based on CLSI interpretation.

# Refer to reference 33.

### Clonal relationship analysis

**Figure 1:**
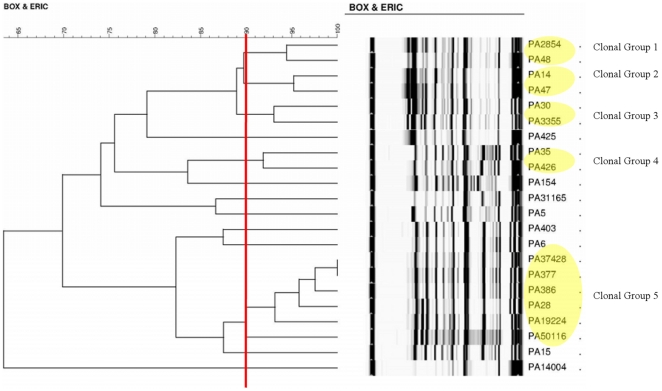
Phylogenetic Tree Diagram showing clonal groups. A yellow oval shape denotes a clonal group after applying a similarity index of ≥90% to PCR typing results.

The phylogenetic dendrogram of the XDR-PA isolates was shown in Figure 1. Applying a similarity index of ≥90% to PCR typing results, 13 distinct clones were identified that included five clonal clusters. Four clusters comprised of two isolates each while the remaining cluster was constituted with 6 isolates. The other eight isolates were clonally unrelated.

### Presence of metallo-beta-lactamase

Fourteen out of 22 XDR-PA isolates tested positive in the imipenem-EDTA double disk synergy test. The presence of MBLs was subsequently confirmed in the same 14 XDR-PA isolates by PCR assay. 50% of the isolates had *bla*_IMP_ alleles while the remaining 50% had *bla*_VIM_ alleles.

### Time-kill Studies

In single antibiotic TKS, none of the antibiotics exhibited bactericidal activity against all isolates at 24 hours. Out of the ten dual-antibiotic combinations tested, meropenem plus PB, amikacin plus meropenem, amikacin plus rifampicin and amikacin plus PB exhibited bactericidal activity in 8/22, 3/22, 1/22 and 6/22 isolates at 24 hours respectively. None of the dual-antibiotic combinations were bactericidal against 10 out of 22 XDR-PA isolates, leading to the testing of triple-drug combinations For these, the amikacin- rifampicin-PB (ARP) combination achieved the highest proportion of bactericidal activity at 24 hours (7 of 10 isolate; 70.0%) followed by the amikacin-meropenem-PB (AMP) (6 of 10 isolate; 60.0%) at 24 hours (Table 2). The AMP and ARP combinations exhibited bactericidal activity against 9 out of 10 isolates in total. None of the three-drug combinations tested displayed bactericidal activity against the remaining isolate (PA 425).

**Table 2:** 24 hour bacteria burden (log_10_ CFU/ml) after exposure to two-drug & three-drug combinations.

		Baseline	Amikacin-Rifampicin-Polymyxin B	Amikacin-Meropenem -Polymyxin B	Meropenem -Polymyxin B	Amikacin-Meropenem	Amikacin-Rifampicin	Amikacin-Polymyxin B
Clonal Group	Isolates	inoculum	Mean	Mean	Mean	Mean	Mean	Mean
6	PA 403	5.10	**0.89**	2.86	3.20	6.86	8.68	6.27
7	PA 154	5.33	-	-	**0.65**	**1.75**	3.27	**1.60**
5	PA 386	5.20	**0.00**	**0.80**	9.00	6.12	8.80	4.49
8	PA 5	5.16	**2.15**	3.39	3.71	3.71	8.09	2.59
9	PA 6	5.76	-	-	3.00	3.16	3.72	**1.24**
10	PA 15	5.47	-	-	4.22	3.86	8.94	**2.12**
3	PA 30	5.19	**1.30**	**0.65**	4.44	4.37	5.13	2.84
4	PA 35	5.55	-	-	**2.26**	8.08	8.76	**1.30**
5	PA 28	4.97	-	-	**1.69**	3.38	8.16	3.55
2	PA 47	5.04	**0.00**	2.83	4.05	5.15	6.54	3.14
1	PA 48	5.08	4.58	**1.45**	2.38	3.28	6.96	7.50
2	PA 14	5.22	**0.00**	**1.00**	5.90	4.11	4.54	3.15
11	PA 14004	5.14	**1.94**	**0.00**	4.49	3.42	8.50	6.50
12	PA 425	5.39	4.19	4.02	4.11	3.70	7.28	4.35
4	PA 426	5.33	-	**-**	**1.60**	4.96	9.12	6.27
5	PA 377	5.14	2.89	**0.00**	4.80	6.23	8.78	4.84
3	PA 3355	5.25	-	-	**1.89**	4.09	4.82	4.35
13	PA 31165	5.00	-	-	3.37	**1.65**	7.27	**0.80**
1	PA 2854	5.58	-	-	**0.00**	6.15	5.19	5.36
5	PA 37428	5.56	-	**-**	**1.95**	8.90	7.47	3.36
5	PA 19224	5.00	-	-	**0.00**	4.43	8.08	6.21
5	PA 50116	5.08	-	-	2.89	**0.00**	**0.00**	**0.00**

Bactericidal combinations denoted in bold.

Comparing the results with respect to the five clonal groups, clonal groups 1, 3 & 5 did not show any similarity for all combinations, while clonal group 2 (PA 47 & PA 14) & clonal group 4 (PA 35 & PA 426) had 50% similarity in the bactericidal triple-drug and dual-drug combinations respectively. (Table 2)

## Discussion

Few treatment options remain for serious infections caused by multidrug-resistant and carbapenem-resistant *P. aeruginosa*. Similarly, options are lacking for XDR *P. aeruginosa* infections. Although clinical trials demonstrating positive outcomes from combination therapy are rare, the use of combination therapy for serious XDR infections is often a case of choosing the devil or the deep blue sea and a last resort in clinically stuck situations.

As compared to colistin (polymyxin E), intravenous polymyxin B has much limited clinical experience across the globe although it is the mainstay in Singapore [20]. However there are a handful of studies that had been conducted during the last 8 years that examine the safety and efficacy of intravenous polymyxin B in nosocomial infections caused by *P. aeruginosa* [9], [21], [22], [23], [24], [25]. In terms of physical chemistry, the only difference between colistin and polymyxin B lies in the replacement of D-Leucine amino acid group in colistin with D-Phenylalanine in polymyxin B [26]. Relating to mechanisms of actions, spectrum of activity and pharmacodynamics, colistin and polymyxin B shared very similar profiles [26], [27].

The combination of an anti-pseudomonal beta-lactam with an aminoglycoside has often been the treatment of choice for this organism. However, this combination does not always prevent *P. aeruginosa* from exhibiting multidrug-resistance phenotypes (especially inducible AmpC-mediated resistance) and clinical failure may still be at risk [28], [29]. The major problem facing the treatment of *P. aeruginosa* infections is the notoriety of the pathogen to possess a wide array of resistance determinants. *P. aeruginosa* can develop resistance to antibiotics either through the expression and/or function of chromosomally encoded mechanisms as a result of mutation or the acquisition of resistance genes on mobile genetic elements (i.e., plasmids) [4]. Multi-drug resistant *P. aeruginosa* are largely mediated by the chromosomal resistance elements that encode for AmpC cephalosporinase, the loss of OprD outer membrane porin, and the multidrug efflux pumps (Resistance Nodulation Division Superfamily Transporter) [5]. Other beta-lactamases such as the PSE beta-lactamases and less common extended-spectrum beta-lactamases (VEB, GES and IBC) are rare and mainly plasmid mediated. OXA-type beta-lactamases had been predominately reported in *P. aeruginosa* as well [30], [31].

MBLs have previously been reported in *P. aeruginosa* clinical isolates in Singapore [32]. In our study, slightly more than half of our XDR-PA isolates were found to harbor MBL-producing genes. All the isolates were highly resistant to all the carbapenems. PA 425 had also been shown to possess VEB-1 like and OXA-10 like genes and PA 5 had OXA-10 genes from a PCR multiplex assay (that screened for MBLs, AmpC, KPC, NDM-1, SHV, TEM, OXA-2, OXA-48, OXA-10 and OXA-18 resistance genes). This might be a possible reason to explain why PA 425 did not respond to any three-drug combination tested. PA 14004 harbours *bla*_VEB-1_ gene and are likely to have overexpressed MexXY efflux pump systems (determined by quantitative real-time reverse transcription-PCR and a 5 fold increase was detected when compared to the ATCC strain) although it was not found to harbor any MBL genes [33]. AMP and ARP are effective bactericidal combinations for PA 14004 while ARP is the only effective bactericidal combination for PA 5. While the rest of the PA isolates do not harbor MBLs, KPC, NDM-1, SHV, TEM, OXA-2, OXA-48, OXA-10 and OXA-18 resistance genes, they are likely to have multidrug efflux pumps and/or the loss of OprD outer membrane porin.

As adequate dosing of antibiotics is pertinent in extremely resistant infections, clinically achievable free or unbound concentrations from maximally possible antibiotic doses were used for all the tested antibiotics to mimic as close as possible the killing effect that takes place in vivo. Since it is the free or unbound protein fraction of a drug that is pharmacologically active, all drug exposures in the experiments were expressed as free drug concentrations [34]. As with all in-vitro models, the main limitation of our study is that the antibiotic concentrations do not fluctuate over time as it does in actual patients. Hence, pharmacodynamic models e.g. hollow-fiber system; in-vivo animal models will be more accurate in mimicking the fluctuating drug-bug interaction along a clinical course, in humans. Furthermore, the usefulness of performing time-kill studies for each clinical isolate is limited by its labor-intensiveness and inability to provide results in a clinically relevant time-frame.

In a fairly similar study by Bergen, *et al* where they examined the activity of colistin in combination with imipenem against a mixture of colistin and imipenem susceptible and resistant strains, colistin heteroresistant and non-heteroresistant strains, and MDR and non-MDR strains of *P. aeruginosa* at different inocula, they reported that colistin combined with imipenem, at increasing clinically relevant concentrations, increased bacterial killing against MDR and colistin-heteroresistant isolates at both high and low inocula [35]. However, they utilized the log change method comparing the change in log10 (cfu/mL) from 0 h (CFU_0_) to time t (6, 24 or 48 h; CFUt), synergism and additivity as the pharmacodynamic endpoints, while we used bactericidal activity (≥3 log_10_ CFU/ml decrease in the colony count from the initial inoculum at 24 hours) as the primary endpoint. This may be attributed to the relatively low colistin MICs that most of the MDR isolates harbored (0.5–2 mg/L), while our XDR-PA isolates had decreased susceptibility to polymyxin B (2–16 mg/L). The key aspects of their study were the use of MDR isolates with varying susceptibilities to colistin and imipenem (including colistin-heteroresistant isolates first identified in this study, and colistin- and imipenem-resistant strains), examination of combinations of clinically relevant drug concentrations at both low and high inocula, and monitoring of emergence of resistance to colistin with real-time population analysis profiles. [35] In addition, only one of their MDR isolates harbors CTX-M and IMP genes, while more than half of our XDR-PA harbor MBL-producing genes. Our primary objective in this study is to identify potential bactericidal antimicrobial combinations that may potentially work against XDR *P. aeruginosa* which exhibit decreasing susceptibilities to polymyxin B. We have also shown that within a clone, different mechanisms of resistance (like those of different carbapenemases) exist and hence, different antibiotics in combination apply.

Few treatment options remain for serious infections caused by XDR *P. aeruginosa* and there had been, to our understanding, no controlled clinical trials to guide therapeutic choices. Until better antibiotics are being developed, novel antibiotic combinations that yield some in-vitro activity are perhaps the best recourse in such scenarios. Due to the varied resistant genotypes that may be exhibited by XDR *P. aeruginosa*, the selection of empirical antibiotic combination therapy may be difficult due to no common effective antibiotic combination for all isolates as well as increased risk of adverse effects posed by the use of triple-agent combinations.

To the best of our knowledge, this is the first study that had objectively evaluated antibiotic combinations for XDR *P. aeruginosa* isolates using the time-kill method and the bactericidal activity as the pharmacological measurement of efficacy. We did not use the conventional (synergistic activity) pharmacological index as our primary measurement of efficacy as all the test isolates were resistant to all the antibiotics (i.e. the synergistic definition may no longer be applicable for XDR *P. aeruginosa* in an useful manner). For example, an XDR *P. aeruginosa* would grow to 8–9 log_10_ CFU/ml, from a baseline inoculum of 5 log_10_ CFU/ml, by 24-hr for a single drug time-kill analysis; the use of a 2-drug combination was synergistic by bringing down the 24-hr inoculum to 6–7 log_10_ CFU/ml. There was still a growth of 1–2 log_10_ CFU/ml, from a baseline inoculum of 5 log_10_ CFU/ml. Bactericidal activity was not observed, despite synergism existed. No clinician would be confident of this synergistic 2-drug combination to give a high likelihood of good clinical outcome.

Although this method cannot make the results available to the clinicians in a timely manner for individual bedside decisions (within 24 to 48 hours), it can help narrow down the possible alternative combinations to use for empiric treatment while waiting for the combination testing to be conducted for every XDR *P. aeruginosa* infection. In addition, we have recently applied the result of this in vitro combination study in a few patients who were infected with similar isolates, as in this study, and good clinical cum microbiological outcomes were observed.

### Conclusion

This study demonstrates the effectiveness of antimicrobial agents when used in combination against *Pseudomonas aeruginosa* isolates with reduced susceptibilities against polymyxin. Our results show that despite documented resistance to various antibiotic classes, bacterial killing may still be achieved with carefully selected antibiotic combinations. Intensive time-kill studies should be reserved for isolates that are resistant against usual effective antibiotic combinations.
